# Pegaspargase Induced Hypertriglyceridemia Resulting in Severe Fatal Pancreatitis

**DOI:** 10.1155/2015/753062

**Published:** 2015-11-29

**Authors:** Neil Vyas, Rafael Ching Companioni, Melik Tiba, Hassan Alkhawam, Aaron Walfish

**Affiliations:** ^1^Department of Medicine, Icahn School of Medicine at Mount Sinai (Elmhurst), 79-01 Broadway, Queens, NY 11373, USA; ^2^Department of Gastroenterology, Elmhurst Hospital Center, 79-01 Broadway, Queens, NY 11373, USA

## Abstract

*Pegaspargase* is used to treat acute lymphocytic leukemia (ALL).* Pegaspargase* definitely has its benefits in treating ALL; however we cannot lose sight of one of its very rare but potentially deadly complications, acute pancreatitis. Clinicians should monitor triglycerides while the patient is on treatment with* Pegaspargase* and suspect acute pancreatitis if the patient develops abdominal pain. If pancreatitis occurs, therapy should be stopped immediately and not reinstituted. For patients with hypertriglyceridemia without pancreatitis, discontinuation of therapy should be considered.

## 1. Case Presentation

**Figure 1:**
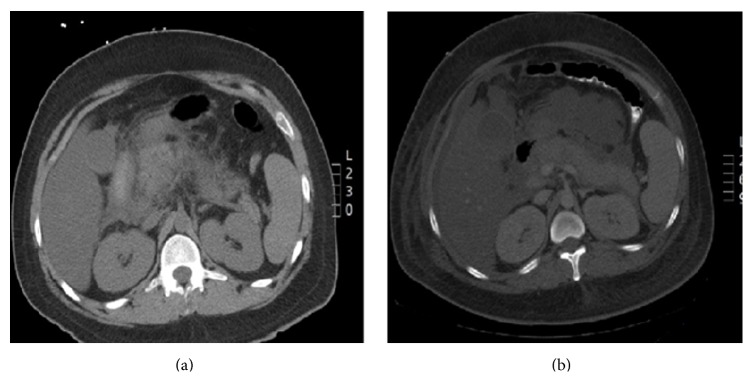
(a) Initial abdomen CT: enlarged pancreas with peripancreatic inflammatory changes consistent with pancreatitis. (b) Interval worsening of the acute pancreatitis. More extensive retroperitoneal fluid extending anteriorly along the psoas muscles more on the right side down to the deep pelvis. Hypoechoic areas in the head of the pancreas/uncinate process possibly areas, perfusion deficits, and parenchymal necrosis. Fluid in the peripancreatic region, lesser sac, and small free intraperitoneal fluid more on the right abdomen.

The patient was a 24-year-old male with a history of Acute T-Cell Lymphoblastic Leukemia (ALL) on recent chemotherapy including* Pegaspargase*. He was admitted to hospital for abdominal pain and was found to have acute pancreatitis secondary to hypertriglyceridemia. Physical examination was significant for tachycardia (127 bpm), decreased air entry in the base of the right lung, and generalized abdominal tenderness and distention. Laboratory tests were remarkable for elevated liver enzymes (ALP 360 U/L, AST 310 U/L, GGT 216 U/L, ALT 44 U/L, and LDH 829 U/L), elevated lipase 228 U/L, and hypertriglyceridemia >3,000 mg/dL. Abdominal CT showed acute pancreatitis with necrosis: peripancreatic, intraperitoneal, and extensive retroperitoneal fluid (Figure 1). Subsequently, his severe pancreatitis was associated with acute kidney injury and respiratory failure, which is illustrated by elevated BUN, creatinine, and persistent hypoxia. According to the Atlanta Classification, patient is classified under severe acute pancreatitis. In addition, patient's BISAP score was 3, which establishes that his risk of death was significantly increasing. Despite appropriate treatment for pancreatitis, according to current guidelines/recommendations, he expired.

## 2. Discussion

The initial chemotherapeutic agent to treat Acute T-Cell Lymphoblastic Leukemia (ALL) was* Asparaginase*. This particular drug underwent extensive testing and was targeted for pegylation, thus forming* Pegaspargase*. The new drug retains its antileukemic effect while allowing less frequent administration.* Pegaspargase* is currently gaining popularity over* Asparaginase* therapy due to having fewer incidences of hypersensitivity reactions and its long half-life (367 hours), allowing dosing every 14 days as opposed to* Asparaginase*, which is dosed daily [1].

*Pegaspargase* (*Oncaspar*) is a modified version of L-asparaginase conjugated with polyethylene glycol. In leukemic cells,* Asparaginase* hydrolyzes L-asparagine to ammonia and L-aspartic acid leading to depletion of asparagine. Leukemia cells need exogenous asparagine; thus asparagine depletion in leukemic cells leads to inhibition of protein synthesis and apoptosis. Despite its potential benefits, there are a wide range of side effects. One rare, but potentially deadly complication, is severe pancreatitis.

Acute pancreatitis is characterized by obstruction of the secretory transport system and activation of pancreatic enzymes. These enzymes autodigest the pancreas leading to inflammation and can lead to impairment of function and morphology. There are several etiologies of pancreatitis, but the two most common causes especially in western countries are alcohol and gallstones. However, there are less common causes such as hyperlipidemia, or more specifically hypertriglyceridemia. The breakdown of triglycerides into toxic free fatty acids (FFA) by pancreatic lipases is the cause of lipotoxicity and ultimately acute pancreatitis in patients with hypertriglyceridemia [2].

In regard to* Pegaspargase*, one proposed mechanism of this drug-induced pancreatitis is hypertriglyceridemia. It is suggested that apolipoprotein E polymorphism may influence the development of hyperlipidemia in ALL patients receiving* Pegaspargase* therapy [3]. One study revealed that apolipoprotein E (apoE) isoform apoE4 (epsilon4) participated in the induction of extreme hypertriglyceridemia, since the frequency of the apoE4/E3 phenotype in the patients with extreme hypertriglyceridemia was higher compared to those in the patients without extreme hypertriglyceridemia and control subjects [4]. Another proposed mechanism is an increase in the apoCIII/apoCII ratio because it functions as an inhibitor and an activator of lipoprotein lipase (LPL), respectively [4]. This ultimately results in an accumulation of triglyceride concentrated lipoproteins in plasma.

Monitoring triglycerides while on* Pegaspargase* therapy would be appropriate in avoiding pancreatitis. Weekly interval monitoring of triglyceride levels will give closer management for patients and prevent hypertriglyceridemia induced pancreatitis. Triglyceride levels of >200 mg/dL–999 mg/dL would be considered moderate and cessation of* Pegaspargase* should be considered and clinically correlated [5]. However, triglyceride levels of >1000 mg/dL are considered a risk factor for pancreatitis and are categorized under severe hypertriglyceridemia [5]; thus cessation of* Pegaspargase* should be instituted. Moreover, when clinical symptoms of pancreatitis such as abdominal pain present, even without hypertriglyceridemia,* Pegaspargase* therapy should be stopped and not reinstituted. There is a time relationship between administering* Pegaspargase *and onset of abdominal pain. Multiple studies have revealed the development of abdominal pain within a median of 2 weeks from the onset of* Pegaspargase *administration [1]. Clinicians should be aware of a higher incidence of pancreatitis with* Pegaspargase *therapy and with careful history taking; focused abdominal examinations and monitoring triglycerides pancreatitis can be avoided.
